# The effectiveness of journals as arbiters of scientific impact

**DOI:** 10.1002/ece3.4467

**Published:** 2018-09-12

**Authors:** C. E. Timothy Paine, Charles W. Fox

**Affiliations:** ^1^ Biological and Environmental Sciences University of Stirling Stirling UK; ^2^ Department of Entomology University of Kentucky Lexington Kentucky USA; ^3^ Present address: Ecosystem Management School of Environmental and Rural Science University of New England Armidale New South Wales Australia

**Keywords:** authorship, citations, manuscript, peer review, publishing, rejection

## Abstract

Academic publishers purport to be arbiters of knowledge, aiming to publish studies that advance the frontiers of their research domain. Yet the effectiveness of journal editors at identifying novel and important research is generally unknown, in part because of the confidential nature of the editorial and peer review process. Using questionnaires, we evaluated the degree to which journals are effective arbiters of scientific impact on the domain of Ecology, quantified by three key criteria. First, journals discriminated against low‐impact manuscripts: The probability of rejection increased as the number of citations gained by the published paper decreased. Second, journals were more likely to publish high‐impact manuscripts (those that obtained citations in 90th percentile for their journal) than run‐of‐the‐mill manuscripts; editors were only 23% and 41% as likely to reject an eventual high‐impact paper (pre‐ versus postreview rejection) compared to a run‐of‐the‐mill paper. Third, editors did occasionally reject papers that went on to be highly cited. Error rates were low, however: Only 3.8% of rejected papers gained more citations than the median article in the journal that rejected them, and only 9.2% of rejected manuscripts went on to be high‐impact papers in the (generally lower impact factor) publishing journal. The effectiveness of scientific arbitration increased with journal prominence, although some highly prominent journals were no more effective than much less prominent ones. We conclude that the academic publishing system, founded on peer review, appropriately recognizes the significance of research contained in manuscripts, as measured by the number of citations that manuscripts obtain after publication, even though some errors are made. We therefore recommend that authors reduce publication delays by choosing journals appropriate to the significance of their research.

## 1 INTRODUCTION

Scholarly journals that peer review submissions provide the primary manner for academic researchers to disseminate their research findings (Rowland, 2002). Journal editors serve as gatekeepers of scholarly publishing, ensuring that research is published in the appropriate location for its quality and significance. They oversee peer review by experts, advising on editorial decisions and providing constructive feedback to improve manuscripts. Peer review gives readers confidence in the validity of the results and interpretation presented in published articles, as those articles have withstood the scrutiny of experts in the field. The peer review process is thus a primary mechanism by which the critical skepticism that characterizes science is put into practice (Ziman, 2000), making it the linchpin of scholarly publishing (Ziman, 1966; Zuckerman & Merton, 1971). Despite the centrality of its role, peer review has been criticized as slowing the process of scientific publishing (Björk & Solomon, 2013; Kareiva, Marvier, West, & Hornisher, 2002; Smith, 1985; Weber, Katz, Waeckerle, & Callaham, 2002), for being expensive, and for its lack of transparency (Kravitz & Baker, 2011). Numerous fixes have been proposed to address these challenging issues (Aarssen & Lortie, 2009; Grossman, 2014; Lortie et al., 2007). More critically, however, the academic publishing system has been criticized for imperfections in judging the quality and significance of research (Goodman, Berlin, Fletcher, & Fletcher, 1994; Lindsey, 1988). Questions about the effectiveness of journals as gatekeepers, that is, as arbiters of scientific quality and significance, remain pressing (Siler, Lee, & Bero, 2015).

Peer review‐based publishing is inherently conservative, as it relies upon the opinions of editors and reviewers, who are themselves selected based on their reputation as scholars (Ziman, 2000; Zuckerman & Merton, 1971). It may thereby stymie the publication of the most creative or unorthodox research: that with the greatest potential to reshape fields of study (Siler et al., 2015). Simultaneously, authors are encouraged to submit their manuscripts to journals with higher impact factor than may be warranted by their quality, in part because many universities use impact factors to rank journals and use these rankings to reward researchers (Ziman, 2000; Zuckerman & Merton, 1971). This has led to a continuing increase in the number of manuscripts submitted to many journals (e.g., Fox & Burns, 2015). Meanwhile, the great disparity among the opinions of reviewers (Fox, 2017) and the lack of incentives for researchers to contribute to peer review (Hochberg, Chase, Gotelli, Hastings, & Naeem, 2009) limit the effectiveness of the peer review process and threaten to reduce the quality of published research. These concerns have led to calls to reform the peer review‐based system of academic publishing (Aarssen & Lortie, 2009; Grossman, 2014; Lortie et al., 2007).

Nevertheless, we have little understanding of the quality of editorial decisions (Ioannidis, 2018). Study of the academic publishing system is complicated by its sensitive and confidential nature. The submission history of manuscripts is rarely public, especially for rejections. Moreover, controlled experiments, such as the simultaneous submission of manuscripts to multiple journals, are disallowed on ethical grounds (Larivière & Gingras, 2010). There is thus a pressing need to evaluate the effectiveness of journals as arbiters of scientific quality.

We surveyed authors of papers published in 146 journals in the research domain of ecology. We queried them on the passage of their manuscripts through the stages of submission, peer review, rejection, and revision, prior to eventual publication. Here, we examine the relationship between editorial decisions and the eventual impact of published papers. We examine the effectiveness of journals as scientific arbiters using three complementary analyses: discrimination against manuscripts of low perceived quality or significance, positive selection of manuscripts that have the greatest scientific impact, and the frequency of mistaken rejections. Higher profile journals are expected to strive to be effective arbiters of scientific influence, given their goal of publishing high‐impact research (Bornmann, Mutz, Marx, Schier, & Daniel, 2011). Therefore, we also assess the degree to which journal performance varies with journal impact factor (JIF). In the absence of a perfect metric of quality and significance for scientific research, we examine the number of citations obtained by a published article. Citations are an imperfect measure of influence because they miss types of impact that do not lead to a reference in a research publication. However, they can be objectively quantified (Rashid, 1991), and they covary with other measures of scientific influence (Mingers & Xu, 2010), including article downloads (Perneger, 2004). Thus, we use the number of citations as the best available indicator of the quality and significance of research, remaining mindful of the fact that they are an imperfect proxy.

## 2 DATA COLLECTION

We obtained metadata, including author details and citations obtained, for all articles published between 2009 and 2015 in 146 journals classified by Clarivate Analytics Web of Science (WoS) in the research domain of ecology. Review and methods journals such as the *Trends* and *Annual Reviews* series and *Methods in Ecology & Evolution* were excluded, yielding 112,515 articles. We additionally obtained the annual journal impact factor (JIF) for each journal and year from WoS Journal Citation Reports.

We sent questionnaires to the corresponding authors of a subsample of these manuscripts. First, we randomly selected 100 articles from every journal by publication–year combination, excluding those classified by WoS as review papers. We then filtered this dataset to include only one randomly chosen article per corresponding author, yielding 38,017 articles. This stratified sampling assured that we had a representative sampling of articles from every journal and publishing year. Second, we extracted articles written by corresponding authors not in the first dataset and again randomly selected one article per corresponding author such that each author was represented only once, yielding an additional 15,579 articles. After excluding duplicated email addresses, 52,543 unique corresponding authors remained.

Using the Qualtrics platform, we sent questionnaires to each corresponding author to request information about the publication history of their paper (Appendix Data 1). We requested details on the history of the published article, including the journals to which the manuscript had been sent, whether it was invited by the journal and the year and outcome of each submission. In total, 12,655 authors, or 24.1% of those contacted, responded to our questionnaire. After removal of incomplete or unintelligible responses, invited manuscripts, and repeated rounds of submission to the same journal, 10,580 questionnaires remained, with a total of 16,981 rounds of manuscript submission.

The data collected through our questionnaire included personal identifiers. Thus, it was essential to maintain the confidentiality of all participants. The manuscript, figures, appendices, and datasets were anonymized to maintain the privacy of authors. CET Paine was the only person with access to data that contained personal identifiers. Human subjects’ ethical approval for this study was obtained from the University of Stirling.

## 3 DESCRIPTIVE RESULTS

**Figure 1:**
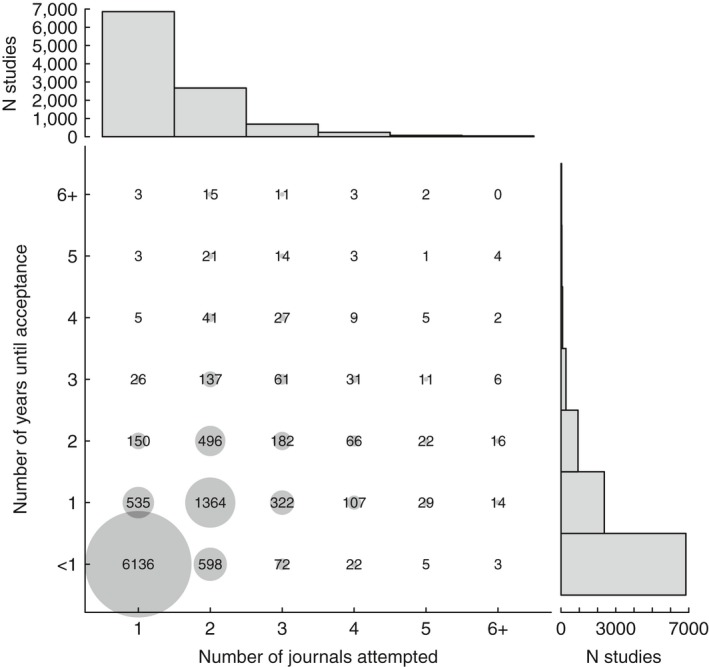
A summary of time to acceptance and number of journals to which manuscripts were submitted. Points are scaled to the number of manuscripts in each category, which is also represented numerically. Distributions are truncated to six journals and six years for presentation. Histograms at the top and right summarize the univariate distributions

Of the 10,580 manuscripts about which authors answered our query, 64.8% were published in the first journal to which they were submitted, whereas those rejected at least once were submitted to a mean of 2.42 journals and took on average 478 days to be published (Figure 1). 13.0% of manuscripts took two or more years from first submission to publication, and 1.6% took four or more years, with the time to publication increasing by 234 days per rejection. 3.4% of manuscripts were rejected from four or more journals.

**Figure A1:**
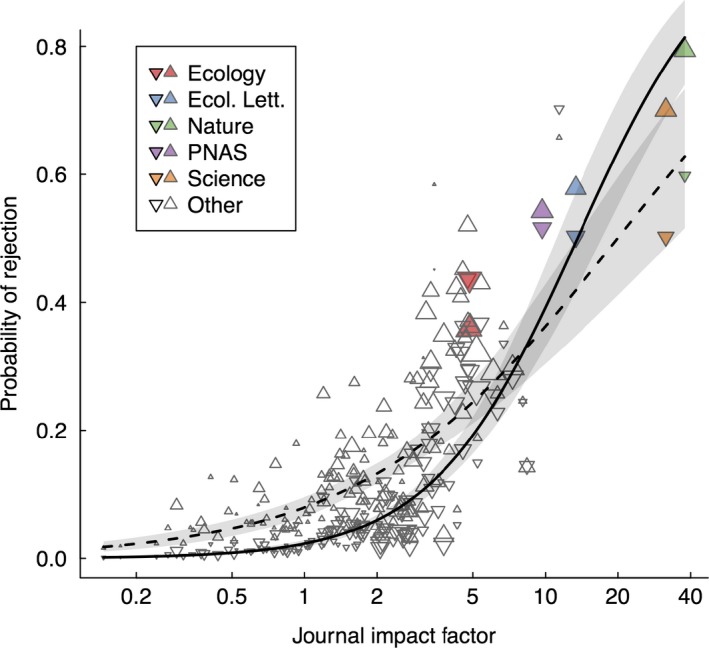
Rejection rates are positively associated with JIF. Points indicate the rejection rate for each journal at the prereview (desk rejection) and postreview stages of the editorial process. Solid line and uptriangles indicate desk rejection, whereas dashed lines and downtriangular points indicate rejection after review. Predictions are derived from a generalized mixed‐effect model with binomial errors and are shown with 95% parametric bootstrap confidence intervals

**Figure A2:**
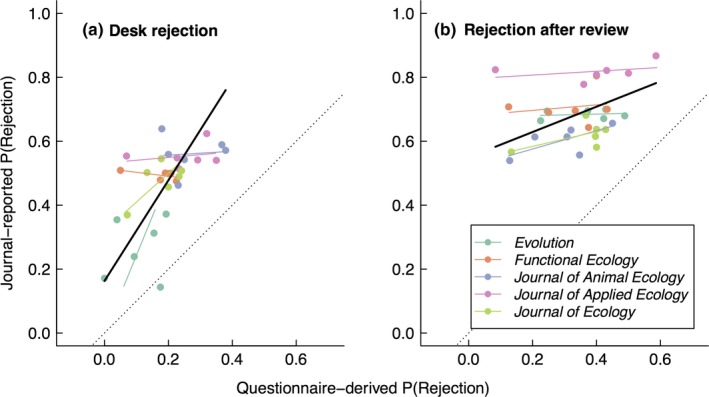
Questionnaire‐estimated rejection rates were positively associated with, but underestimated, true rejection rates in the five journals for which data were available (major axis regression; desk rejection: *p* = 0.0009; rejection after review: *p* = 0.07). Points indicate the annual rejection rates per journal in each year from 2010 to 2015. Dotted lines indicate the 1:1 relationship. Heavy black lines represent the overall major axis regression relationships, whereas lighter, colored lines show the relationships fit for each journal

In our questionnaire response sample, the median rate of rejection among journals was 15.7% (interquartile range (IQR): 7.6%–31.2%). Fourteen journals accepted all submissions and 17 rejected >50% of the manuscripts (Appendix Table A1). Overall, rejection rate was strongly positively associated with journal impact factor (Appendix Figure A1). Rejection after review was more frequent than desk rejection at most journals, but this reversed at journals with JIF greater than nine (Generalized linear mixed‐effect model: *p* < 0.0001). Questionnaire‐derived rejection rates were positively associated with, but consistently underestimated, true (journal‐reported) rejection rates for the five journals for which independent estimates of rejection rates were available (major axis regression; desk rejection: *p* = 0.0009, rejection after review: *p* = 0.07; Appendix Figure A2); author‐reported rejection rates were 58%–71% of true rejection rates.

## 4 ANALYTICAL METHODS AND RESULTS

To determine the effectiveness of journals as arbiters of scientific significance, that is, how well they screen out low‐impact papers and accept high‐impact papers, we need a metric of the significance of both accepted and rejected papers. However, manuscripts rejected by a particular journal are (by definition) never published by that journal. This means that the effects of rejection on the number of citations obtained are confounded with the effects of constructive feedback from the editor and reviewers and the influence of the publishing journal (Larivière & Gingras, 2010). Moreover, rejected manuscripts are published later, following additional rounds of resubmission and review. Finally, there is a “halo effect” of publishing in prominent journals, which stems, in part, from the tendency of authors to disproportionately cite articles from higher impact journals (Merton, 1968). Thus, it is difficult to directly compare the counts of citations between accepted and rejected manuscripts. We do so using four complementary metrics, the performance ratio, the gatekeeping ratio, the rejection rates of future high‐impact studies, and the frequency of mistaken rejections (summarized in Table 1). To reduce heteroscedasticity, these metrics were log‐transformed prior to analysis. Analyses were conducted in R 3.4.4 (R Core Team 2018). Significance tests and confidence intervals were determined through parametric bootstrapping, implemented in packages lme4 and pbkrtest (Bates, Mächler, Bolker, & Walker, 2015; Halekoh & Højsgaard, 2014).

**Table 1:** Summary of metrics used to assess the effectiveness of journals as scientific arbiters. Although this study focuses on studies published in 146 journals in the domain of ecology, some analyses included more journals, as many of the studies we examined had been rejected from journals outside that set

Metric	Formula	*N* journals	*N* mss
Performance ratio	$\frac{1\;+\;N\;{\text{citations}}_{\mathrm{rejected}\;\mathrm{by}\;k,\mathrm{published}\;\mathrm{by}\;j}}{1\;+\;\mathrm{median}({N\;\mathrm{citations}}_{\mathrm{first}\;\mathrm{intents},j})}$	450	3,594
Gatekeeping ratio	$\frac{1\;+\;\text{median}(N\;{\text{citations}}_{\mathrm{published}\;\mathrm{by}\;j)}}{1\;+\;(N\;{\text{citations}}_{\mathrm{rejected}\;\mathrm{by}\;k,\mathrm{published}\;\mathrm{by}\;j)}}$	443	3,525
Rejection ratio	$\frac{P{\left(\mathrm{rejection}\right)}_{\mathrm{run}-\mathrm{of}-\mathrm{the}-\mathrm{mill},k,y}}{P{\left(\mathrm{rejection}\right)}_{\mathrm{high}-\mathrm{impact},k,y}}$	98	2,236
Proportion of rejected manuscripts that became that obtained more citations than the median paper in the rejecting journal	$\frac{N\;{\text{rejected}\;\text{mss}}_{\mathrm{cittaions}>\mathrm{median}{\left(\mathrm{citations}\right)}_{k}}}{{N\;\mathrm{rejected}\;\mathrm{mss}}_{k}}$	310	3,160
Proportion of rejected manuscripts that became high‐impact papers in the publishing journal	$\frac{N\;{\text{rejected}\;\text{mss}}_{\mathrm{citaions}>90\mathrm{th}\;\mathrm{percentile}{\left(\mathrm{citations}\right)}_{k}}}{{N\mathrm{rejected}\;\mathrm{mss}}_{k}}$	142	3,778

Note

*j*: publishing journal; *k*: rejecting journal; *y*: publishing year; *mss*: manuscripts.

### 4.1 Performance ratio

#### 4.1.1 Methods

The first aspect of scientific arbitration is the degree to which journals identify and reject low‐quality manuscripts. If a journal is an effective arbiter of scientific impact, the manuscripts they reject should go on to gain fewer citations, once published in a different journal, than papers published in that journal and year that had not previously been rejected. A mean “performance ratio” for manuscripts rejected from a particular journal of <1.0 indicates that that journal, on average, correctly rejects low‐impact manuscripts and is therefore an effective scientific arbiter (Table 1).

To assess this aspect of scientific arbitration, we first calculated, for every combination of journal and publishing year, the median number of citations obtained by studies that were published by that journal that had not previously been rejected from another journal (i.e., “first intents”, Calcagno et al., 2012). Medians capture the central tendency of the skewed distribution of citation numbers for articles within journals better than do means. We then calculated, for each manuscript that had previously been rejected from another journal, the ratio of the number of citations it obtained to the median number of citations for first‐intent manuscripts at the relevant publishing journal and year. We added one to the numerator and denominator to avoid divide‐by‐zero errors for journals where the median number of citations was zero. Because both the numerator (citations obtained by previously rejected papers) and denominator (median citations obtained by papers not previously rejected) are calculated including only papers for which we had survey responses, any nonresponse bias toward (or against) high‐impact papers should not influence this metric. Because we expected that the degree of underperformance of previously rejected papers would covary with difference in JIF between the rejecting journal and the publishing journal, we predicted this “performance ratio” as a function of the percentage change in JIF between journals in a linear mixed‐effect model. The percentage change in JIF between journals was binned into categories of < −60%, −60% to −20%, −20% – +20%, and > +20%. The model included rejecting journal and publishing year as random effects.

#### 4.1.2 Results

**Figure 2:**
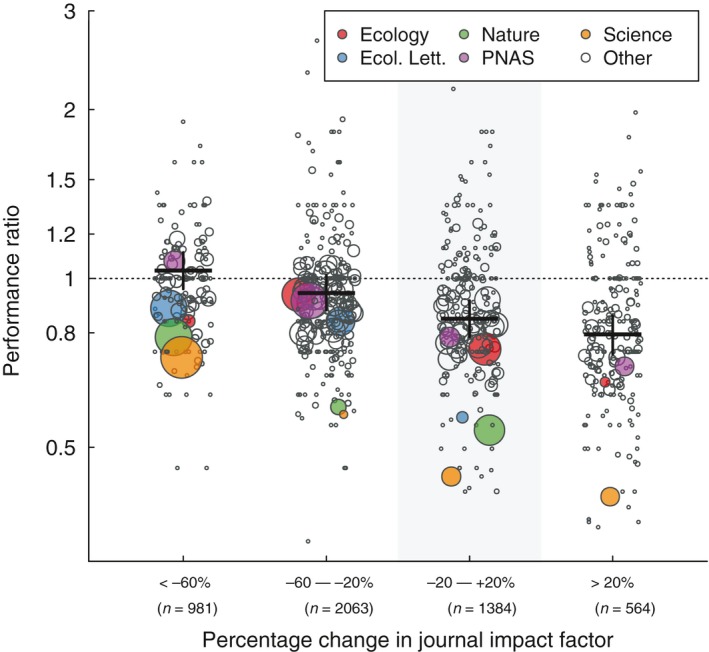
Manuscripts that are rejected from one journal and resubmitted to another tend to gain fewer citations than those accepted at the first journal to which they were submitted (i.e., “first intents”). The “performance ratio” of resubmitted manuscripts was lowest for manuscripts sent to journals of a greater impact factor than the original journal. The shaded region highlights manuscripts that were resubmitted to a journal of similar JIF to that from which they were rejected. Mean estimates are shown with 95% confidence intervals, obtained by parametric bootstrapping. Points indicate the mean performance ratio for each rejecting journal and are sized according to the number of manuscripts rejected by each journal in each category. Selected prominent journals are indicated with colored symbols. The number of manuscripts in each percentage change category is shown. All groups differ significantly in performance ratio (Tukey's honest significant difference, *p* ≤ 0.0051)

78.0% of rejected manuscripts were resubmitted to journals of lower JIF. Manuscripts that were resubmitted to a journal of similar or higher JIF obtained 16.3% and 21.9% fewer citations, respectively, than the median study published in the same year and journal that had not previously been rejected (Figure 2). Papers resubmitted to journals with 20‐60% lower JIF than the rejecting journal performed equivalently to other manuscripts published in that same journal and thus appear to have found the right outlet for their significance. Papers resubmitted to substantially lower JIF journals, on the other hand, obtained 3.3% more citations than did direct submissions (*p* < 0.0001; statistical details of all analyses are available in Appendix Table A2). These results indicate that manuscripts rejected by editors generally underperform (in terms of number of citations) other papers had they not been rejected and thus that journals disproportionately reject manuscripts with reduced potential to advance their field of study, compared to those they accept.

### 4.2 Gatekeeping ratio

#### 4.2.1 Methods

The performance ratio, described above, quantifies the degree to which previously rejected manuscripts underperform first intents (manuscripts published in the first journal to which they are submitted) within a *publishing* journal. Next, we examine the degree to which editors are appropriately rejecting low‐impact manuscripts. Specifically, we asked whether papers rejected from each individual journal *k* generally go on to under or overperform the average paper in their final publication outlet, *j*, and tested how this varied across journals. We assessed this by calculating a “gatekeeping ratio,” which is similar to the performance ratio (Figure 2) in that previously rejected papers are compared to papers published in the same final journal, but differ in that the gatekeeping index averages relative performance of papers across submissions to the rejecting journal, rather than across submissions to the publishing journal. We calculated the median number of citations obtained by all articles published in journal *j* in year *y*, divided by the number of citations obtained by manuscript *i* rejected from journal *k* and published in journal *j* in year *y* (Table 1). We calculated the gatekeeping ratio of every manuscript that experienced a rejection prior to publication. We estimate the gatekeeping ratio for journal *k* by taking the mean over manuscripts rejected by that journal. A “gatekeeping ratio” of 1.0 for a journal indicates that papers it rejected go on to be just as well cited as does the median paper in the journal in which they are eventually published. Ratios > 1.0 indicate that manuscripts rejected by the journal obtain fewer citations than the median paper published in their final outlets, with higher ratios indicating a greater difference.

We predicted the gatekeeping ratio of each rejected manuscript as a function of the editorial stage at which it was rejected (pre‐ or postreview) and tested how it varied with the impact factor of the rejecting journal, using a linear mixed‐effect model that allowed for random variation among rejecting journals. The magnitude of the gatekeeping ratio is also affected by the difference in prominence between journals *j* and *k*. We account for this difference by including the log‐transformed ratio of their JIFs as a predictor in the model. There was no evidence for an effect of rejection stage (i.e., desk rejection versus postreview rejection) on gatekeeping ratio (*p* = 0.28), so we deleted that term and its interaction with JIF from the models.

#### 4.2.2 Results

**Figure 3:**
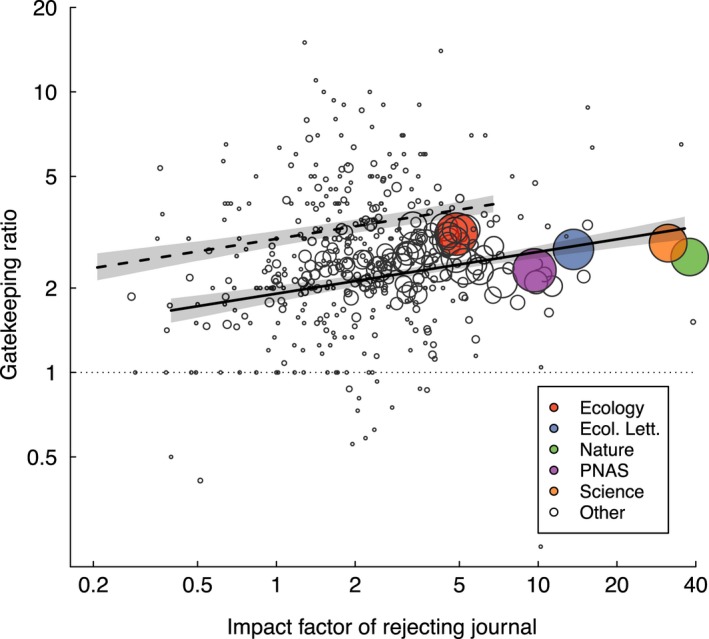
The effectiveness of journals as scientific arbiters, as measured through the “gatekeeping ratio,” increased with the impact factor (JIF) of the rejecting journal. Each point is one journal mean, with point size scaled to the number of manuscripts evaluated. Regression lines are shown with 95% confidence intervals. The solid line indicates rejected manuscripts resubmitted to journals with JIF half of the rejecting journal, whereas the dashed line indicates rejected manuscripts resubmitted to journals with JIF twice that of the rejecting journal. The horizontal dotted line indicates a gatekeeping ratio of 1.0, meaning that rejected manuscripts obtain the same number of citations as the average paper in their publishing journal

Papers that had been rejected underperformed the median paper in the publishing journal for 93% of journals (Figure 3). Manuscripts that were rejected gained just 40.3% as many citations (averaged across rejecting journals) as the median paper published in the publishing journal (IQR: 29.2%–52.4%). The number of citations obtained by rejected manuscripts was negatively associated with the change in JIF between rejecting and publishing journals (*p* < 0.0001), meaning that papers that cascade further down in JIF rankings between submissions underperform less, consistent with the performance ratio results (Figure 2). Regardless of the change in JIF, however, gatekeeping ratios were consistently >1.0.

Gatekeeping ratio was positively associated with JIF, meaning that papers rejected from higher JIF journals underperformed papers published in the publishing journal more strongly than did papers rejected from lower JIF journals (*p* < 0.0001; Figure 3).

### 4.3 Ability to identify high‐impact studies

#### 4.3.1 Methods

An effective scientific arbiter would be sensitive to, and more likely to accept for publication, high‐impact manuscripts that go on to make greater contributions to the field of study than do solid but run‐of‐the‐mill manuscripts (Siler et al., 2015). In contrast to the previous analyses, here we focus on the ability of journals to detect the most impactful studies. We categorized submissions to each journal in each year into citation quantiles as “run‐of‐the‐mill” or “high‐impact,” depending on whether they received more than the 90th percentile of citations received by manuscripts published in that journal in that year. This categorization was performed separately for each journal and year, making it independent of the rejection rates of journals and accounting for the nonlinear accumulation of citations through time. Journal‐by‐year combinations for which fewer than ten manuscripts were available in our dataset were excluded. We predicted the probability of manuscript rejection as a function of citation quantile and editorial stage (desk rejection or rejection after review), allowing for random variation in the effect of citation quantile among journals, in a generalized mixed‐effect model. Residuals were assumed to follow a binomial distribution, as manuscript rejection is a binomial (reject/not reject) process.

To test the assertion that high‐JIF journals are more effective scientific arbiters than are less prominent journals, we predicted the rejection rate for every combination of journal, citation quantile, and editorial stage from a generalized linear mixed‐effect model. To do so, we calculated the “rejection ratio” for each journal at each stage as the estimated rejection rate for future run‐of‐the‐mill papers divided by the estimated rejection rate for future high‐impact papers (Table 1). A ratio of 1.0 indicates that a journal was equally likely to reject “high‐impact” and “run‐of‐the‐mill” manuscripts, with increased effectiveness indicated by larger values. Finally, we predicted the rejection ratio as a function of journal impact factor and editorial decision (desk rejection or rejection after review) in a weighted linear regression. Weights were the number of manuscripts submitted to each journal, to account for variation in the number of submissions among journals. Note that this analysis does not assess how gatekeeping effectiveness may vary through time. Accordingly, we used a time‐averaged impact factor for each journal. There was no evidence of different JIF rejection ratio relationships between stages, so we simplified the model by deleting the interaction.

#### 4.3.2 Results

**Figure 4:**
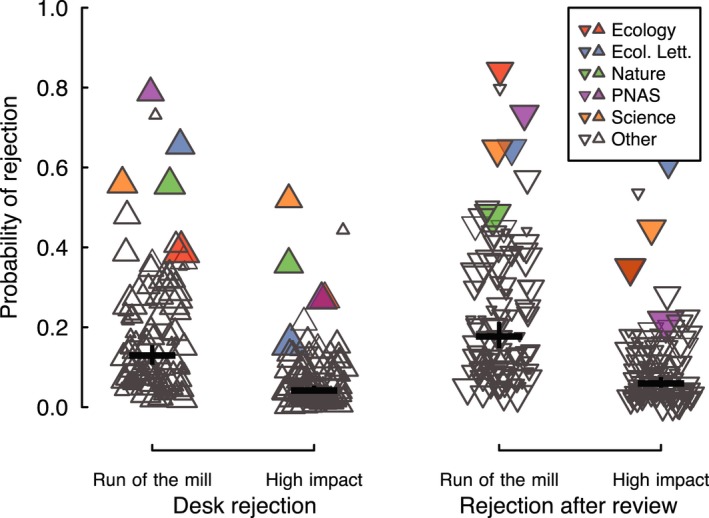
Most journals were more likely to reject run‐of‐the‐mill manuscripts than high‐impact manuscripts. High‐impact manuscripts are defined as the 10% of manuscripts that obtained the most citations in each individual journal, and run‐of‐the‐mill manuscripts are the remaining 90%. Mean estimates are shown with 95% confidence intervals, obtained by parametric bootstrapping. Each point represents a journal, with point sizes scaled by the number of manuscripts evaluated

Overall, manuscripts that went on to become high‐impact papers were significantly less likely to have been rejected, both before (desk rejection) and after review, than were manuscripts that became run‐of‐the‐mill papers (Figure 4; *p* < 0.0001). By this metric, journals were twice as effective as scientific arbiters at the desk rejection stage than after review (*p* < 0.0001). At the average journal, high‐impact manuscripts were 23.0% as likely to have previously been desk‐rejected than were run‐of‐the‐mill manuscripts, but 41.1% as likely to have been rejected after review. This indicates that editors are less likely to reject future high‐impact papers than they are to reject future run‐of‐the‐mill papers, but that they have a fairly high error rate, especially after review. Journals varied significantly in the rate at which they rejected high‐impact studies, as indicated by a significant interaction between publishing journal and citation quantile (*p* = 0.0001).

**Figure 5:**
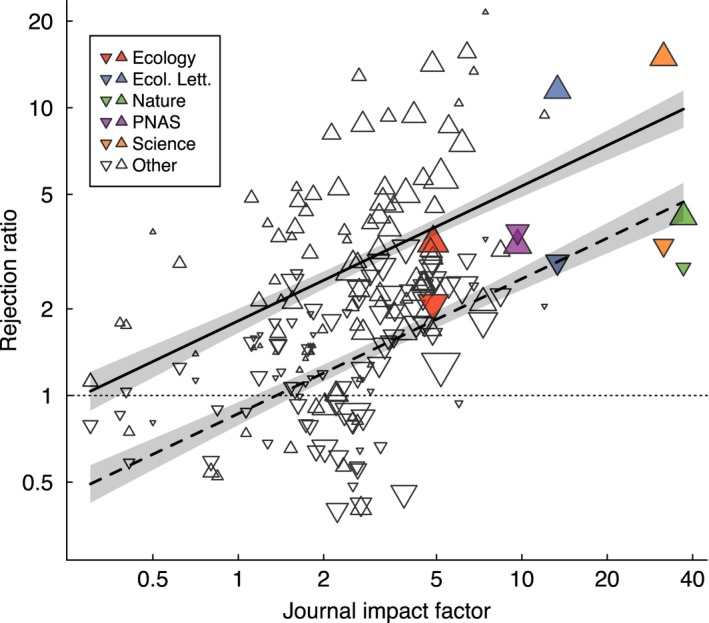
Sensitivity to high‐impact research, as measured through the “rejection ratio,” increased with journal impact factor (JIF) and was greater before review (i.e., desk rejection; solid line and uptriangular points) than postreview (dashed line and downtriangular points). Each point represents one journal mean and is sized according to the number of manuscripts evaluated at each editorial stage. Regression lines are derived from a linear model, which was weighted by the number of manuscripts evaluated by each journal and are shown with 95% confidence intervals. Horizontal dotted line indicates a rejection ratio of 1.0, corresponding to journals that are equally likely to reject “high‐impact” and “run‐of‐the‐mill” manuscripts

**Figure A3:**
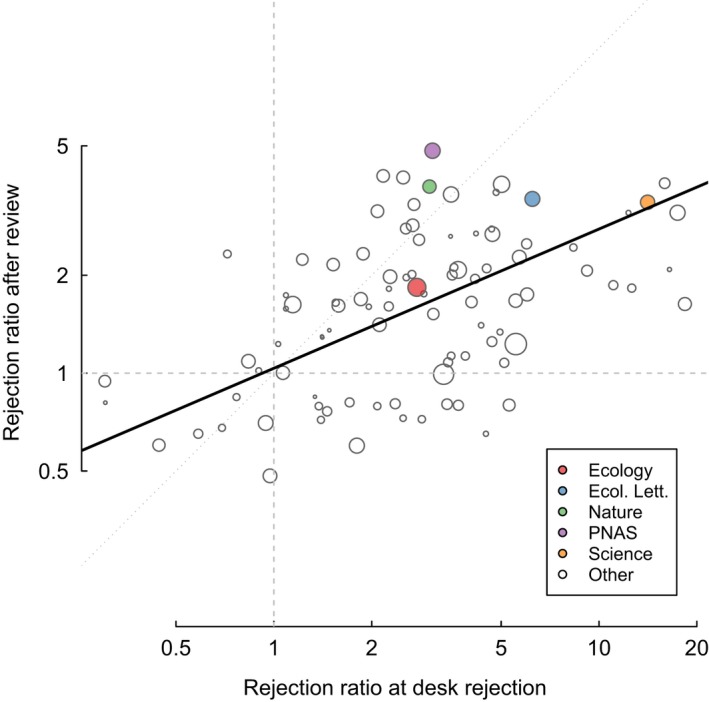
Rejection ratios before and after review are positively associated (major axis regres sion: *p* < 0.0001). Solid black line represents the overall major axis regression. Dashed gray lines indicate a rejection ratio of 1, which corresponds to journals that are equally likely to reject “run‐of‐the‐mill” or “high‐flyer” manuscripts. Dotted gray line indicates the 1:1 relationship

High‐JIF journals were generally better at distinguishing future high‐impact papers than low‐JIF journals (*p* < 0.0001; Figure 5). High‐JIF journals were much more likely to accept high‐impact manuscripts relative to run‐of‐the‐mill manuscripts, whereas the disparity in rejection rates between these two types of manuscripts was smaller at low‐JIF journals. Doubling JIF was associated with a 24% increase in a journal's rejection ratio. The strength of the relationship between JIF and rejection ratio did not differ between editorial decision stages (*p* = 0.36; Figure 5). By this metric, 74% of journals examined could be considered effective arbiters of science, in that they were less likely to reject future high‐impact papers than future run‐of‐the‐mill papers both before and after review. 18% of journals were ineffective at distinguishing these two types of papers at one stage or the other, and 6% were ineffective gatekeepers at both stages (Appendix Figure A3). Notably, some of the most prominent journals were no more likely to accept high‐impact manuscripts than were substantially less prominent journals (Figure 5).

### 4.4 Frequency of mistaken rejections

#### 4.4.1 Methods

As a final metric of scientific arbitration, we define two types of errors in manuscript handling by journals: the rejection of manuscripts that go on to (a) obtain more citations than the median paper published in the rejecting journal or (b) become high‐impact papers in the publishing journal. For the former analysis, we considered only rejected manuscripts that were resubmitted to journals of lower JIF. If such manuscripts outperform their original journal, they are likely to be true mistakes, as they have had to overcome a decrease in journal prominence. However, the possibility that authors have increased the quality of their manuscript through substantial revision cannot be discounted. In both analyses, we predicted the frequency of mistaken rejection as a function of the JIF of the rejecting journal using generalized linear models with a binomial error distribution.

#### 4.4.2 Results

**Figure 6:**
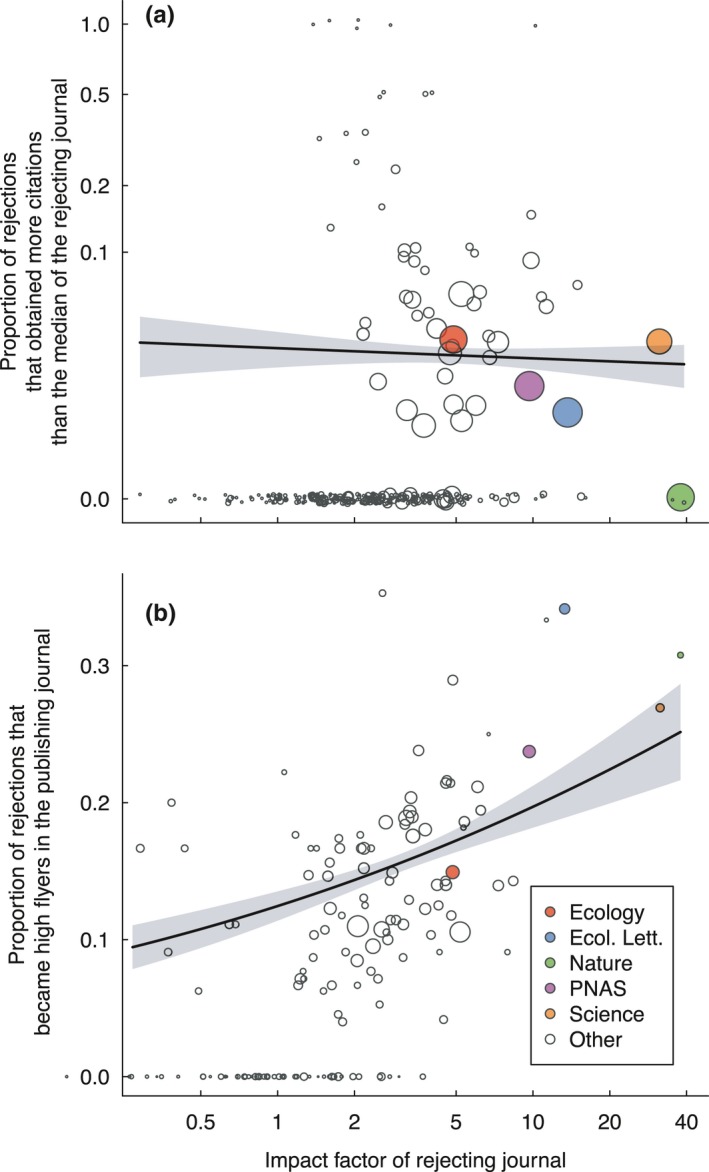
The frequency of mistaken rejections per journal, as assessed by (a) the proportion of rejected manuscripts that attain more citations than the median article in the rejecting journal or (b) the proportion of rejected manuscripts that go on to be high‐impact papers in the publishing journal. Each point represents one journal mean and is scaled according to the number of manuscripts evaluated. Mean estimates are shown with 95% confidence intervals, obtained by parametric bootstrapping. Note that in panel A, the y‐axis is on a log scale, and points are slightly jittered to improve visualization

3.8% of rejected manuscripts went on to become papers that gained more citations than the median article in the journal that rejected them, whereas 9.2% of rejected manuscripts went on to become high‐impact papers in the publishing journal (Figure 6). Thus, both types of rejection mistakes were infrequent. The frequency of the first type of error was independent of JIF, whereas the frequency of the second type increased with increasing JIF (generalized linear models: *p* = 0.685 and *p* = 0.0012, respectively).

## 5 DISCUSSION

Interacting with the academic publishing system imposes a steep burden on researchers, who struggle with rejections of their manuscripts, potentially from multiple journals. They tolerate it because publication in peer‐reviewed journals is linked to professional rewards, including hiring, promotion, and research funding (Merton, 1968; Ziman, 2000; Zuckerman & Merton, 1971). The continuing acceptance of the academic publishing system depends, at least in part, on the understanding that editors effectively sort manuscripts into outlets appropriate to their level of impact and that they help to improve manuscript quality (Ioannidis, 2018). Our results indicate that the journals we investigated distinguish, by and large, the quality of the studies they receive and publish and are therefore effective arbiters of scientific quality. Our results thus counsel for moderation in efforts to reform the system of academic publishing (Aarssen & Lortie, 2009; Grossman, 2014; Lortie et al., 2007). Previous studies of journals as arbiters of research impact have examined at most a few journals (Bornmann et al., 2011; Braun, Dióspatonyi, Zsindely, & Zádor, 2007; Siler et al., 2015; Zuckerman & Merton, 1971), whereas our study includes all 146 of the Web of Science‐indexed journals in the domain of ecology. We therefore expect our results to apply widely across academic publishing.

Journals varied tremendously in the degree to which they screened out low‐impact manuscripts and identified high‐flyer manuscripts. On average, high‐JIF journals were more likely to reject manuscripts that underperform (in citations) an average paper published in that same journal (Figure 3). They were also less likely to reject high‐impact studies (Figure 5). The success of high‐JIF journals at identifying high‐impact papers may be because they generally have large editorial boards, allowing them to assign papers to editors with the most appropriate experience for the papers being evaluated. Similarly, their prominence likely allows them to recruit higher profile editors and reviewers, the experience of whom presumably makes high‐JIF journals better able to identify studies of great scientific merit. High‐JIF journals likely also receive more papers that are inappropriately targeted to the wrong tier of journal, because of benefits accruing to authors that successfully publish in a high‐impact journal (Ziman, 2000; Zuckerman & Merton, 1971), potentially making the most important and significant manuscripts more likely stand out.

Additionally, although journals generally share the aim of publishing high‐quality science, not all aim to selectively publish high‐impact science; it is likely that the degree to which journals try to be gatekeepers of research significance varies with JIF. Some journals, such as *PLoS ONE* and *Ecology and Evolution*, aim to publish all competently executed studies, rather than restricting themselves to studies of outstanding quality or impact. A further consideration is that only studies in ecology were examined in this study. This research domain is a minor component of multidisciplinary journals such as *Nature*,* PLoS ONE*,* PNAS*, and *Science*. Therefore, the implications of this study for such journals should be interpreted cautiously. Further studies could contrast the effectiveness of for‐profit and not‐for‐profit publishers and assess gatekeeping effectiveness in terms of a journal's support for reproducible research (Ioannidis, 2005). Overall, our analyses strongly support the interpretation that high‐JIF journals are effective, but not perfect, at discerning high‐impact research from less significant research.

What is the role played by editors and reviewers in scientific gatekeeping? The gatekeeping ratio—the degree to which previously rejected papers underperform the average manuscript published in their final outlet—did not differ between editorial stages (i.e., pre‐ and postreview), but the rejection ratio—a measure of the ability to distinguish high‐flying manuscripts—was greater before than after review (Figures 4 and 5). If editors were able to identify high‐impact manuscripts before review and thus sent only the most promising manuscripts out for review, we would expect the rejection ratio to be negatively associated between editorial stages because there would be less variance in quality among manuscripts sent for review, making it more difficult to select among those manuscripts. We see no support for such a trade‐off among journals (Appendix Figure A3). In fact, there was a positive relationship; journals that had a high rejection ratio at the desk rejection stage also tended to have a high rejection ratio following review (major axis regression: *p* < 0.0001). This suggests that editors and reviewers contribute differently to the gatekeeping role of journals. Editors desk reject the weakest papers, shape the remit of their journals by rejecting papers that are out of scope, and shepherd studies that they see as having great potential through peer review (Rowland, 2002; Smith, 1985). Reviewers, on the other hand, contribute by distinguishing among, and helping to improve, the mass of average‐quality manuscripts (Bakanic, McPhail, & Simon, 1987; Goodman et al., 1994), but likely play a lesser role in distinguishing high‐impact manuscripts from run‐of‐the‐mill ones. High‐JIF journals desk reject a greater proportion of manuscripts (Appendix Figure A1) and more selectively discriminate between high‐impact and run‐of‐the‐mill papers than do less prominent journals (Figure 5). Although the causality of this relationship is not easy to decipher, it suggests that the relative importance of editors in assessing manuscript impact increases with JIF, relative to that of reviewers, consistent with a recent simulation study (Esarey, 2017).

**Figure A4:**
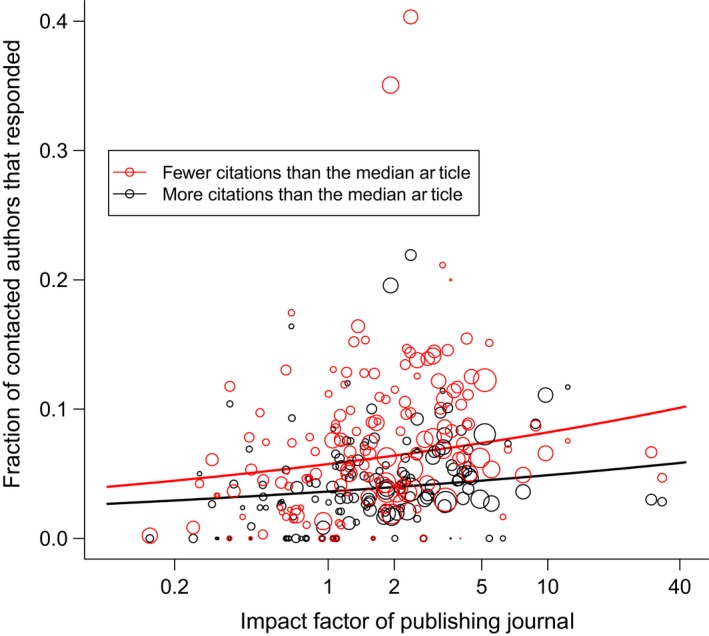
Response rates of authors to the manuscript history questionnaire, as predicted by the impact factor of the publishing journal and whether the published paper obtained more citations than the median paper published in the same journal and year. Authors were more likely to respond to the questionnaire if their papers were published in higher JIF journals and gained fewer than the median number of citations. These contrasting patterns suggest that nonresponse bias was minor and therefore had little effect on our study

Author‐reported rejection rates, assessed through questionnaires, underestimated true, journal‐reported, rejection rates in our study (Appendix Figure A4). There are several possible explanations for this. First, questionnaires were only sent to authors whose research was published in journals indexed by Web of Science (WoS). Thus, authors whose papers were published in nonindexed journals, or not published at all, were never sent questionnaires and are thus omitted from the current study. If we assume the rejected papers that were published in nonindexed journals or were never published are those of the lowest quality, then our analyses underestimate gatekeeper effectiveness, as the lowest impact factor papers are not included in our dataset. It is possible that this bias is greatest for low‐JIF journals, as there are fewer WoS‐indexed outlets of lower JIF available for manuscripts rejected from such journals. Additionally, authors whose manuscripts were rejected prior to publication may have been less likely to respond to the questionnaire than were authors of manuscripts that were accepted at one of the first journals they targeted.

We investigated the potential for nonresponse bias by evaluating the rate of response to our questionnaire as a function of the JIF of the publishing journal and whether the published paper gained more citations than the median for papers published in the same journal and year. Authors were more likely to respond to the questionnaire if their papers were published in higher JIF journals, but also if their paper gained *fewer* than the median number of citations (generalized linear mixed‐effect model: *p* < 0.0001; Appendix Figure A4). These two patterns run counter to each other—higher response rates for higher impact papers among journals, but lower response rates for higher impact papers within journals– such that their effects on outcomes could largely counteract each other. There are two further reasons to expect that nonresponse bias was minor. First, only authors who were ultimately successful in publishing their manuscript in an indexed journal were contacted. Eventual publication lessens the sting of rejection (Clapham, 2016). Second, many authors described in their responses the difficulties they had experienced in the publication of their manuscript. Although we cannot confirm that nonresponse biases would have no effect on our results, such biases would need to be large to obscure the main effects we report; we are thus confident that the results we present are reliable.

Authors appeared receptive to the feedback received from journals, in that the great majority of resubmissions were sent to lower JIF journals (Figure 2). Controlling for publication year and rejecting journal, resubmitted manuscripts were less well cited than were papers accepted at the first journal they targeted, regardless of whether they were resubmitted to a higher or lower JIF journal (Figures 2 and 3). However, the degree of underperformance varied with the change in JIF between submissions; papers resubmitted to higher JIF journals tended to underperform most (Figure 2). These results are not driven by the delay incurred in revision and resubmission, as our analysis controlled for publication year. Our finding is consistent with a study of chemistry manuscripts (Bornmann & Daniel, 2008), but stands in contrast to two recent studies that found that resubmitted manuscripts received more citations (Calcagno et al., 2012; Siler et al., 2015). The studies of Bornmann and Daniel (2008) and Siler et al. (2015) examined <2,000 manuscripts each and only a handful of journals, whereas our sample size is much larger (*N *=* *10,580 manuscripts). The result of Calcagno et al. (2012), on the other hand, although statistically significant owing to their extraordinarily large dataset, had a minute effect size, in contrast to the substantial effect sizes observed here (Appendix Table A2). We are therefore confident that for a diverse set of journals, resubmitted manuscripts are less cited than are those accepted at the first targeted journal.

One possible explanation for the underperformance of previously rejected papers is that authors commonly target for their first submission journals with higher JIF than is warranted from the significance of their manuscript, leading to rejection (Figures 2 and 3). Moreover, when their paper is rejected, some authors persist in resubmitting to overly high‐JIF journals, likely explaining why some manuscripts are repeatedly rejected (Figure 1). Such papers are then poorly cited in their final publication outlet, relative to submissions that did not submit to overly high‐JIF journals and, thus, were published in the first journal to which they were submitted. This interpretation implies that some authors disregard the message sent by editors who declined their paper, either explicitly in editorial comments or implicitly by the decision made (i.e., that the quality or significance of the paper is not appropriate for such a high‐JIF journal). Many authors may also fail to revise their manuscripts following rejection in a manner that increases their quality or significance, which is unfortunate, given the evidence that peer review improves scientific articles (Bakanic et al., 1987; Goodman et al., 1994).

Our results suggest that researchers often submit their manuscripts to journals that for which they are unsuitable, but that editors and reviewers are generally good at identifying and rejecting such papers (Figures 3 and 4). Choosing the wrong outlet for one's scholarly manuscript, especially when editorial advice is ignored, can lead to repeated rejection and substantially delayed publication. It also imposes substantial costs on editors and reviewers, due to the need for repeated reconsideration of the same manuscripts. The amount of work involved in reviewing and rejecting a manuscript is similar to that of reviewing and accepting it (Rowland, 2002). In theory, researchers should know their study better than any editor or reviewer and thus should be uniquely well equipped to judge its quality. In reality, however, authors often appear to overestimate the significance of their own work (Wynder, Higgins, & Harris, 1990). It also may be that the rewards accruing to authors who publish at a high‐impact journal outweigh the costs (delays in publication if rejected). That many investigators persist in resubmitting to journal of similar impact factor despite multiple rejections suggests that both mechanisms are at work, contributing to the crisis of reviewing that confronts scientific publishing (Hochberg et al., 2009). Reducing the number of low‐quality manuscripts flung at high‐JIF journals would make it less difficult to find peer reviewers (Fox, 2017; Fox, Albert, & Vines, 2017), accelerate scientific publishing, and potentially forestall a decline in the quality of published research (Higginson & Munafò, 2016).

Despite the well‐publicized flaws in peer review‐based academic publishing, our results show that journals are effective at identifying the most impactful research from the diversity of submissions that they receive and are therefore effective scientific gatekeepers.

## DISCLOSURE STATEMENT

C. E. T. Paine is an associate editor at Functional Ecology and at Biotropica, and C. W. Fox is an executive editor at Functional Ecology. Both of these journals were examined in this study.

## AUTHOR CONTRIBUTIONS

CETP and CWF conceived the study, and CETP collected the data and led the analyses with contributions from CWF. CETP and CWF wrote the manuscript together.

## DATA ACCESSIBILITY

Data available from the Dryad Digital Repository: https://doi.org/10.5061/dryad.6nh4fc2.

## APPENDIX 1 Data A1: Questionnaire

### 1.1

Please answer the following questions with reference to the initial submission of the manuscript. Dear Dr $Last_Name: I am conducting a study of the fluxes of manuscripts among scientific journals in the biological sciences, and one of your articles has been selected: $Authors. $Year_Published}. $Title. $Publication_Name} $Volume:$Beginning_Page‐$Ending_Page. The questionnaire will inquire about each time you submitted the manuscript that led to this paper to a scientific journal. For the purposes of the questionnaire, please consider every round of review separately. In other words, if your manuscript went through multiple rounds of review at a single journal, report each of them separately.
*Why participate?* You could win one of three £50 Amazon gift certificates, to be awarded to three randomly selected participants at the end of the data‐collection period. This study will generate recommendations to authors, editors and publishers to maximize the likelihood that any given manuscript will result in a highly cited paper. Your participation will help to increase the quality of submitted manuscripts and the peer‐review process.
*Privacy, independence and ethics* The data collected in this study include personal identifiers. Thus, it is essential to maintain the confidentiality of all participants. I take your privacy very seriously. I will be the only person with access to data that contains personal identifiers. The information you provide will be used solely for the purpose of this study. Any data that I share with collaborators will be stripped of all personally identifiable markers, such as names, addresses, institutions, and the titles of manuscripts. This study is independent of the journals and publishers. They have had no role in the study design or execution. Nor will they have any role in the analysis, reporting or interpretation of the results. I have obtained human‐subjects ethical approval for this study from the University of Stirling. Please complete the questionnaire with one month, by Sunday, August 14th. If you have any questions or concerns about this study please contact me directly. Additionally, if you feel that one of your co‐authors would be better able to complete the questionnaire, please forward this email to them. Thank you very much for your cooperation!

1. Which was the FIRST journal where this manuscript was submitted?
2. In what year did you initially submit this manuscript for publication?
3. What was the outcome of your initial submission?
  - Desk rejection (i.e., rejection without peer review)
  - Peer review, then rejection
  - Peer review, then rejection with invitation to resubmit
  - Peer review, then request for major revisions
  - Peer review, then request for minor revisions
  - Peer review, then accepted for publication
  - Accepted for publication without peer review
  - Withdrawal by author
  - Other (please specify) ____________________
4. How many peer reviews were performed on your initial submission? (free text)
5. Rate the overall quality of the peer reviews performed on your initial submission: (Excellent, Good, Average, Poor, Terrible)
6. Please provide any further comments about your initial submission (Optional)

*NOTE*: Questions 1‐6 repeated for every subsequent round of submission, until submissions were marked as “Accepted for publication without peer review.”

1. Please rank the importance of factors that led you to select this/these scientific journal(s) to publish your study:

**Table:**

Importance	Prestige of journal	Alignment of journal with the topic of manuscript	Impact factor of journal	Open‐access policy of journal	Suggestion of journal editor or reviewer	Suggestion of academic supervisor
Extremely						
Very						
Moderately						
Slightly						
Not at all						

7a Please mention any other factors that led you to select this/these scientific journal(s) to publish your study (optional, free text)
8 Please tick the options that best describe your opinions about the overall process of publishing this study.

**Table:**

Agreement	Publishing this manuscript was challenging	Comments from peer reviewers improved the manuscript	Comments from editors improved the manuscript	The published article was of higher quality than the initial manuscript
Strongly agree				
Agree				
Somewhat agree				
Neither agree nor disagree				
Somewhat disagree				
Disagree				
Strongly disagree				

8a Please provide any further comments about the overall process of publishing this study (optional, free text)
9 Indicate your gender: (Male, Female)
10 How old were you when you wrote the manuscript? (Less than 25, 25‐35, 36‐45, 46‐55, More than 55)
11 What was the highest academic degree you had attained when you wrote the manuscript? (High school [Pre‐university], Bachelors [B.A., B.Sc], Masters [M.A., M.Sc., Mres], Doctoral [Ph.D., D.Phil, M.D.])
12 How many peer‐reviewed scientific articles had you written (as lead or contributing author) prior to the study in question? (0 [this was my first article], 1‐2, 3‐5, 6‐10, 10 or more)
13 Rate your English‐language fluency at the time you wrote the manuscript: (Native speaker, Very fluent, Somewhat fluent, Slightly fluent, No knowledge of English)

### 1.2

**Table A1:** Overview of the 146 journals classified by Web of Science in the research domain of ecology. We present the number of manuscripts examined, the overall rejection percentage, the percentage of rejection before review (desk rejection), and rejection after review, calculated from author responses to our survey. We also present the performance of each journal in terms of our four metrics scientific arbitration. Empty cells indicate journals for which too few data were available to calculate the relevant statistic

Journal	2015 Impact factor	N mss submitted	Rejection rate (%)				Rejection ratio		Rejection mistakes	
			Overall	Prereview	Postreview	Gatekeeping ratio	Prereview	Postreview	% mss with *N* citations > mean *N* citations of publishing journal	% mss that would have been high flyers in rejecting journal
*Acta Amazonica*	0.408	13	30.77	0.00	30.77					0.00
*Acta Oecologica*	1.420	72	36.11	11.11	25.00	2.30	9.49	1.82	0.00	71.88
*African J Ecology*	0.875	78	19.23	5.13	14.10	1.59	0.73	2.12	0.00	83.33
*African J Range & Forage Sci*.	1.250	13	7.69	7.69	0.00					0.00
*Agriculture Ecosystems & Envt*.	3.564	198	16.16	5.56	10.61	2.65	6.68	1.55	0.00	81.58
*American Midland Naturalist*	0.592	78	14.10	6.41	7.69	1.48	4.22	1.03	0.00	66.67
*American Naturalist*	3.148	261	55.94	14.94	41.00	3.30	2.24	2.02	0.00	87.50
*Animal Conservation*	2.788	88	37.50	19.32	18.18	2.61	15.17	1.79	0.00	82.14
*Annales Zoologici Fennici*	0.753	25	12.00	4.00	8.00	1.57				0.00
*Applied Ecol. & Envtl. Research*	0.500	31	19.35	6.45	12.90	2.18				0.00
*Applied Vegetation Science*	2.308	52	25.00	5.77	19.23	3.50	5.03	2.49	0.00	39.13
*Aquatic Ecology*	1.797	45	22.22	4.44	17.78	2.32	1.69	1.19	0.00	31.25
*Aquatic Invasions*	1.955	60	16.67	3.33	13.33		3.48	1.61		16.67
*Aquatic Microbial Ecology*	2.109	65	9.23	3.08	6.15	2.12	2.35	1.48	0.00	26.67
*Austral Ecology*	1.598	102	16.67	4.90	11.76	3.25	5.01	1.77	0.00	69.44
*Basic & Applied Ecology*	1.836	68	26.47	8.82	17.65	2.48	5.96	1.34	0.00	53.85
*Behavioral Ecology*	3.029	190	33.68	9.47	24.21	1.90	1.51	2.12	10.71	88.89
*Behavioral Ecol. & Sociobiology*	2.382	193	23.32	2.59	20.73	2.32	3.01	3.59	0.00	81.43
*Biochemical Systematics & Ecol*.	0.988	63	9.52	0.00	9.52	1.68	0.83	0.65		77.78
*Biodiversity & Conservation*	2.258	165	25.45	19.39	6.06	2.53	5.49	0.77	3.85	83.33
*Biogeosciences*	3.700	238	4.20	2.10	2.10	1.43	2.17	0.53	0.00	87.76
*Biological Conservation*	3.985	351	46.15	25.93	20.23	2.80	4.87	2.98	1.03	81.97
*Biological Invasions*	2.855	209	21.05	11.96	9.09	2.48	3.94	1.03	0.00	85.11
*Biology Letters*	2.823	277	41.88	8.66	33.21	2.74	5.09	3.11	9.09	81.03
*Biotropica*	1.944	147	31.29	11.56	19.73	2.54	11.07	2.00	4.76	83.33
*BMC Ecology*	2.724	15	13.33	6.67	6.67	3.00			0.00	0.00
*Bull. Peabody Mus. Nat. Hist*.	1.217	11	0.00	0.00	0.00					0.00
*Chemistry & Ecology*	1.281	34	5.88	0.00	5.88					0.00
*Chemoecology*	1.863	11	27.27	9.09	18.18	3.00				0.00
*Community Ecology*	1.019	33	15.15	0.00	15.15	1.93	1.78	1.25	0.00	35.71
*Compost Sci. & Utilization*	0.564	14	7.14	0.00	7.14					0.00
*Conservation Biology*	4.267	228	53.51	33.33	20.18	2.93	1.39	1.89	0.00	95.83
*Conservation Letters*	7.126	54	27.78	16.67	11.11	2.23	0.95	1.14	10.00	45.45
*Contemporary Prob. of Ecology*	0.259	10	0.00	0.00	0.00					0.00
*Diversity & Distributions*	4.566	167	47.31	26.95	20.36	2.84	2.74	2.49	3.33	78.38
*EcoMont*	0.278	7	14.29	0.00	14.29					0.00
*Ecography*	5.355	181	48.07	28.18	19.89	3.11	4.00	1.66	2.38	82.00
*Ecohydrology*	2.138	59	5.08	1.69	3.39	2.50	3.00	0.66	0.00	61.11
*Ecological Applications*	4.252	249	44.18	26.91	17.27	2.37	5.72	2.21	4.41	86.00
*Ecological Complexity*	1.797	28	7.14	0.00	7.14					0.00
*Ecological Economics*	3.227	200	10.00	2.50	7.50	2.16	0.84	0.77	0.00	88.57
*Ecological Engineering*	2.740	156	3.21	0.00	3.21	5.41	0.53	0.56		90.00
*Ecological Informatics*	1.683	30	3.33	0.00	3.33					0.00
*Ecological Modelling*	2.275	194	13.40	3.61	9.79	2.03	1.11	0.96	33.33	84.78
*Ecological Monographs*	8.037	61	52.46	26.23	26.23	1.89	14.79	3.04	4.00	50.00
*Ecological Research*	1.338	58	22.41	1.72	20.69	2.93	1.31	1.69	0.00	37.93
*Ecology*	4.733	463	57.02	30.02	27.00	3.11	2.72	1.86	3.91	85.07
*Ecology & Evolution*	2.537	200	4.00	1.00	3.00	1.75	1.17	0.45		88.44
*Ecology Letters*	10.772	344	70.64	42.73	27.91	2.75	6.18	3.43	1.33	65.85
*Ecoscience*	0.595	32	18.75	3.13	15.63	5.00	1.69	1.20	0.00	38.46
*Ecosphere*	2.287	149	11.41	0.67	10.74	3.48	0.30	0.70	0.00	90.48
*Ecosystem Services*	4.307	33	6.06	3.03	3.03		1.69	1.19		18.18
*Ecosystems*	3.751	128	17.19	2.34	14.84	3.43	3.07	2.51	0.00	76.19
*Ecotoxicology*	2.329	72	6.94	2.78	4.17	6.00	2.50	0.72		57.89
*Ecotropica*	0.350	10	0.00	0.00	0.00					0.00
*Ekoloji*	0.592	9	0.00	0.00	0.00					0.00
*Environmental Biology of Fishes*	1.404	99	11.11	3.03	8.08	2.24	2.61	1.01	0.00	85.71
*European J Soil Biology*	1.951	38	10.53	0.00	10.53	3.50				0.00
*European J Wildlife Research*	1.403	96	16.67	5.21	11.46	1.99	5.21	1.34	0.00	70.59
*Evolution*	4.007	310	50.32	12.26	38.06	2.57	21.04	3.10	1.54	71.05
*Evolutionary Ecology*	1.875	70	18.57	7.14	11.43	2.39	3.07	1.80	0.00	64.29
*Evolutionary Ecology Research*	0.896	26	7.69	3.85	3.85	2.60				0.00
*Fire Ecology*	1.098	21	4.76	0.00	4.76					0.00
*Flora*	1.590	76	13.16	1.32	11.84	1.60	1.86	1.50	0.00	57.14
*Freshwater Science*	2.433	80	12.50	2.50	10.00	3.86	3.20	1.84	0.00	76.47
*Frontiers in Ecol. & Envt*.	8.504	47	38.30	21.28	17.02	2.35	19.78	2.03	0.00	36.36
*Functional Ecology*	5.210	179	50.84	18.44	32.40	3.22	1.19	2.26	0.00	88.24
*Fungal Ecology*	2.631	46	8.70	0.00	8.70		1.31	1.46		42.11
*Global Change Biology*	8.444	353	45.04	15.01	30.03	2.09	0.99	1.80	3.80	86.05
*Global Ecology & Biogeography*	5.840	144	38.89	20.83	18.06	2.63	19.18	3.46	6.67	80.56
*Heredity*	3.801	81	24.69	3.70	20.99	2.97	4.31	2.03	0.00	68.97
*Interciencia*	0.219	22	9.09	4.55	4.55	3.00				0.00
*Intl. J Sust. Devel. & World Ecol*.	1.609	26	0.00	0.00	0.00					0.00
*ISME J*	9.328	154	24.03	3.90	20.13	1.78	3.73	1.50	0.00	82.86
*Israel J Ecology & Evolution*	0.727	5	20.00	20.00	0.00					0.00
*Journal for Nature Conservation*	2.220	47	10.64	4.26	6.38	4.00	0.28	0.75	0.00	40.91
*J Animal Ecology*	4.827	200	58.50	28.50	30.00	3.09	3.48	2.77	0.00	78.57
*J Applied Ecology*	5.196	259	65.64	26.25	39.38	2.91	1.91	1.94	3.23	78.57
*J Arid Environments*	1.623	108	13.89	7.41	6.48	2.78	5.64	1.13	0.00	93.33
*J Biogeography*	3.997	196	33.16	14.80	18.37	2.94	2.79	3.47	0.00	85.71
*J Biological Dynamics*	1.147	9	0.00	0.00	0.00					0.00
*J Chemical Ecology*	3.151	87	9.20	2.30	6.90	2.06	2.06	0.77	0.00	64.71
*J Ecology*	6.180	242	57.85	19.01	38.84	3.23	22.13	1.68	1.20	81.40
*J Evolutionary Biology*	2.747	216	38.43	5.56	32.87	2.91	7.04	1.56	0.00	80.65
*J Exptl. Marine Biology & Ecology*	1.796	232	6.90	0.86	6.03	1.91	1.14	0.67	0.00	91.53
*J Fish & Wildlife Management*	0.795	33	3.03	0.00	3.03					0.00
*J Freshwater Ecology*	0.720	38	10.53	5.26	5.26	2.71	1.69	1.20	0.00	18.75
*J Natural History*	1.010	93	5.38	0.00	5.38	5.67	0.70	0.62		93.33
*J Plant Ecology*	1.769	27	14.81	14.81	0.00	3.57			0.00	0.00
*J Tropical Ecology*	0.975	103	33.01	6.80	26.21	2.59	5.87	0.91	0.00	93.33
*J Vegetation Science*	3.151	132	37.88	13.64	24.24	2.46	3.83	2.54	0.00	82.86
*J Wildlife Management*	1.725	181	23.20	2.21	20.99	2.53	2.61	3.63	0.00	85.37
*Landscape & Ecol. Engineering*	0.597	3	33.33	0.00	33.33	6.50				
*Landscape & Urban Planning*	3.654	120	10.83	5.00	5.83	4.36	4.11	0.75	0.00	100.00
*Landscape Ecology*	3.657	106	28.30	14.15	14.15	1.98	13.33	1.76	0.00	77.42
*Marine Biology Research*	1.649	59	13.56	8.47	5.08	2.44	5.39	0.59	0.00	41.18
*Marine Ecology Progress Series*	2.361	444	11.04	2.70	8.33	2.68	4.02	0.96	0.00	89.25
*Microbial Ecology*	3.232	74	9.46	2.70	6.76	5.24	3.43	0.69	0.00	52.17
*Molecular Ecology*	5.947	370	43.24	6.49	36.76	2.30	10.79	2.14	1.45	78.85
*Molecular Ecology Resources*	5.298	7	100.00	14.29	85.71	1.16			0.00	
*Natural Areas Journal*	0.626	48	12.50	4.17	8.33	3.83	2.71	1.67		38.89
*Nature*	38.138	291	85.57	73.54	12.03	2.58	2.96	3.72	0.00	69.23
*NZ J Ecology*	1.247	24	8.33	4.17	4.17					0.00
*Northeastern Naturalist*	0.569	63	3.17	0.00	3.17		1.08	0.93		50.00
*Northwest Science*	0.412	31	6.45	0.00	6.45		1.69	1.20		36.36
*Oecologia*	2.902	314	49.04	24.20	24.84	2.58	4.58	2.95	1.32	81.11
*Oikos*	3.586	213	53.05	25.82	27.23	3.33	1.84	2.33	6.00	79.63
*Oryx*	2.052	89	15.73	6.74	8.99	5.37	4.67	1.07	0.00	82.35
*Paleobiology*	2.959	41	9.76	2.44	7.32	4.83				0.00
*Pedobiologia*	1.535	39	2.56	0.00	2.56					0.00
*Persp. Plant Ecol. Evol. & Syst*.	3.578	27	25.93	7.41	18.52	1.40			50.00	0.00
*Phytocoenologia*	1.828	16	0.00	0.00	0.00					0.00
*Plant Ecology*	1.490	124	17.74	5.65	12.10	2.60	4.62	1.06	12.50	84.21
*Plant Species Biology*	1.303	17	5.88	5.88	0.00	3.00			0.00	0.00
*PLoS Biology*	8.668	61	83.61	72.13	11.48	2.03	4.57	3.49	5.71	66.67
*PLoS ONE*	3.057	184	25.00	5.43	19.57	3.03	6.87	3.01	5.00	82.43
*Polar Biology*	1.711	118	11.86	2.54	9.32	3.24	2.84	0.78	0.00	69.57
*Polar Record*	0.904	31	0.00	0.00	0.00		1.60	0.78	0.00	28.57
*Polar Research*	1.728	23	0.00	0.00	0.00					0.00
*Polar Science*	1.157	4	0.00	0.00	0.00					0.00
*Polish J Ecology*	0.500	31	0.00	0.00	0.00					0.00
*Polish Polar Research*	1.182	11	0.00	0.00	0.00					
*Population Ecology*	1.698	47	21.28	4.26	17.02	3.04	4.20	2.45	0.00	12.00
*Proc. Acad. Nat. Sci. Philly*	0.952	8	12.50	0.00	12.50	1.75				0.00
*Proc. Linnean Soc. NSW*	0.214	4	0.00	0.00	0.00					
*Proc. Nat. Acad. Sci. USA*	9.423	352	69.60	45.74	23.86	2.31	3.04	4.67	2.03	76.27
*Proc. Royal Soc. B*	4.823	612	45.75	14.05	31.70	2.39	5.68	1.25	6.42	89.44
*Rangeland Ecology & Mgmt*.	1.349	65	26.15	4.62	21.54	2.10	5.62	2.68	0.00	41.67
*Rangeland Journal*	1.194	32	18.75	0.00	18.75					0.00
*Restoration Ecology*	1.891	119	26.05	6.72	19.33	3.16	7.21	2.36	0.00	90.91
*Revista Chilena de Hist. Nat*.	0.738	30	3.33	0.00	3.33					0.00
*Russian J Ecology*	0.456	22	0.00	0.00	0.00					0.00
*Science*	34.661	327	77.06	55.35	21.71	2.89	14.27	3.36	3.67	73.08
*South African J Wildlife Research*	1.641	17	5.88	0.00	5.88					0.00
*Southeastern Naturalist*	0.513	73	5.48	1.37	4.11	1.73	1.65	0.72		75.00
*Southwestern Naturalist*	0.255	92	10.87	5.43	5.43	1.86	3.97	0.64		83.33
*Theoretical Ecology*	2.085	32	15.63	0.00	15.63	2.71			0.00	0.00
*Theoretical Population Biology*	1.452	32	18.75	3.13	15.63	3.97				0.00
*Tropical Ecology*	1.169	25	8.00	0.00	8.00					0.00
*Urban Ecosystems*	1.984	64	3.13	0.00	3.13		0.92	0.77		68.75
*Vie et Milieu*	0.222	12	8.33	0.00	8.33					0.00
*Western N. Am. Naturalist*	0.291	66	7.58	1.52	6.06	1.41	1.68	0.69	0.00	54.55
*Wetlands*	1.504	108	19.44	1.85	17.59	2.40	1.75	0.78	0.00	82.61
*Wildlife Biology*	0.745	26	26.92	11.54	15.38	2.00			0.00	0.00
*Wildlife Monographs*	5.125	5	60.00	40.00	20.00	14.00				0.00
*Wildlife Research*	1.000	66	16.67	3.03	13.64	2.10	5.21	1.29	0.00	28.57

mss: manuscripts.

#### Table A2 Statistical details for all analyses

All *p*‐values and confidence intervals for mixed‐effect models were determined through parametric bootstrapping.

*Figure 2: Performance Ratio*

Linear mixed effect model fit by lme4::lmer
Formula: Performanceratio ~ JIF.pc.cat + (1 | Year.end) + (1 | Journal.start)

Random effects:
Groups Name Variance Std.Dev.
 Journal.start (Intercept) 0.007177 0.08471
 Year.end.f (Intercept) 0.008196 0.09053
 Residual 0.139743 0.37382
Number of obs: 4903, groups: Journal.start, 443; Year.end.f, 6
Fixed effects:
 Estimate Std. Error t value CI.2.5% CI.97.5% P‐value
(Intercept) 0.03220 0.04023 0.800 ‐0.044 0.0108 0.200
JIF.pc.cat‐60 ‐ ‐20% ‐0.09196 0.01624 ‐5.663 ‐0.122 ‐0.0600 <0.0001
JIF.pc.cat‐20 ‐ +20% ‐0.19767 0.01726 ‐11.456 ‐0.230 ‐0.1640 <0.0001
JIF.pc.cat> +20% ‐0.26193 0.02142 ‐12.229 ‐0.303 ‐0.2191 <0.0001

*Figure 3: Gatekeeping Ratio*

Linear mixed effect model fit by lme4::lmer
Formula: GKratio_pub_med ~ JIF.start.l + JIF.lr + (1 | Journal.start)
Random effects:
Groups Name Variance Std.Dev.
 Journal.start (Intercept) 0.001791 0.04233
 Residual 0.109608 0.33107
Number of obs: 4029, groups: Journal.start, 443
Fixed effects:
 Estimate Std. Error t value CI.2.5% CI.97.5 P‐value
(Intercept) 0.37855 0.01258 30.091 0.35194 0.40382 < 0.0001
JIF.start.l 0.14890 0.02080 7.159 0.10594 0.19092 < 0.0001
JIF.lr 0.32425 0.02267 14.300 0.27848 0.36747 < 0.0001

*Figure 4: Probability of Rejection*

Generalized linear mixed model fit by lme4::glmer, binomial family with logit link
Formula: cbind(Reject, Accept) ~ stage * quantile + (quantile | Journal)
Random effects:
 Groups Name Variance Std.Dev. Corr
 Journal (Intercept) 1.4342 1.1976
 quantileHigh‐flyer 0.3301 0.5746 ‐0.86
Number of obs: 388, groups: Journal, 97
Fixed effects:
 Estimate Std. Error z value CI.2.5% CI.97.5% P‐value
(Intercept) ‐2.01422 0.12968 ‐15.532 ‐2.2759 ‐1.7611 < 0.0001
stageR 0.33121 0.03838 8.629 0.2558 0.4068 < 0.0001
quantileHigh‐flyer ‐1.23901 0.15804 ‐7.840 ‐1.5573 ‐0.9306 < 0.0001
stageR:quantileHigh‐flyer 0.68844 0.14800 4.652 0.4013 0.9847 < 0.0001

*Figure 5: Rejection Ratio*

Weighted linear model fit by stats::lm
Formula: log_Rejectionratio ~ JIF.start.l + stage, weights = n
Coefficients:
 Estimate Std. Error t value CI.2.5% CI.97.5% P‐value
(Intercept) 0.22825 0.04073 5.604 0.14790 0.30858 < 0.0001
JIF.start.l 0.49921 0.05649 8.838 0.38779 0.61062 < 0.0001
stageR ‐0.30857 0.03698 ‐8.345 ‐0.38151 ‐0.23563 < 0.0001

*Figure 6: Frequency of mistakes*

*Panel A: Rejections that obtain more citations than the mean of the rejecting journal*

Generalized linear model fit by stats::glm
Formula: Prop_mistake ~ JIF.start.l, weights = n, binomial family with logit link
Coefficients:
 Estimate Std. Error z value CI.2.5% CI.97.5 P‐value
(Intercept) ‐2.886 0.162 ‐17.767 ‐3.210 ‐2.573 < 0.0001
JIF.start.l ‐0.091 0.226 ‐0.406 ‐0.541 0.346 0.685

*Panel B: Rejections that became high flyers in the publishing journal*

Generalized linear model fit by stats::glm
Formula: cbind(High_flyer, Run_of_the_mill) ~ JIF.start.l, binomial family with logit link

Coefficients:
 Estimate Std. Error z value CI.2.5% CI.97.5% P‐value
(Intercept) ‐1.9503 0.0977 ‐19.948 ‐2.1148 ‐1.7615 < 0.0001
JIF.start.l 0.5450 0.1675 3.252 0.2170 0.8741 0.00115
